# Suggestion, persuasion and work: Psychotherapies in communist Europe

**DOI:** 10.1080/13642537.2017.1421986

**Published:** 2018-01-03

**Authors:** Sarah Marks

**Affiliations:** ^a^ Department of History, Classics and Archaeology, Birkbeck, University of London, London, UK

**Keywords:** Communism, suggestion, history of psychotherapy, work therapy, Soviet Union, Eastern Europe, sugestión, comunismo, Europa del Este, Unión soviética, terapia de trabajo, historia de la Psicoterapia, Europa orientale, Unione Sovietica, Suggestione, Comunismo, Terapia del lavoro, Storia della psicoterapia, Europe de l’Est, Union Soviétique, suggestion, communisme, thérapie par le travail, histoire de la psychothérapie, Ανατολική Ευρώπη, Σοβιετική Ένωση, Πρόταση, Κομμουνισμός, Θεραπεία μέσω εργασίας, Ιστορία της ψυχοθεραπείας

## Abstract

This article traces what recent research and primary sources tell us about psychotherapy in Communist Europe, and how it survived both underground and above the surface. In particular, I will elaborate on the psychotherapeutic techniques that were popular across the different countries and language cultures of the Soviet sphere, with a particular focus upon the Cold War period. This article examines the literature on the mixed fortunes of psychoanalysis and group therapies in the region. More specifically, it focuses upon the therapeutic modalities such as work therapy, suggestion and rational therapy, which gained particular popularity in the Communist countries of Central and Eastern Europe. The latter two approaches had striking similarities with parallel developments in behavioural and cognitive therapies in the West. In part, this was because clinicians on both sides of the ‘iron curtain’ drew upon shared European traditions from the late nineteenth and early twentieth centuries. Nevertheless, this article argues that in the Soviet sphere, those promoting these approaches appropriated socialist thought as a source of inspiration and justification, or at the very least, as a convenient political shield.

## Abstract (es)

Este artículo rastrea lo que las investigaciones recientes y fuentes fidedignas nos dicen acerca de la psicoterapia en la Europa comunista y cómo ésta sobrevivió en dos formas: abiertamente y en la clandestinidad. Particularmente me referiré a las técnicas psicoterapéuticas que fueron populares a través de los diferentes países y lenguajes culturales de la esfera soviética con especial foco en el período de la ‘’guerra fria’’. Se examina la literatura acerca de la suerte desigual del psicoanálisis y de las terapias de grupo en la región, concentrándonos específicamente en algunas modalidades como la terapia de trabajo, la sugestión y la terapia racional, las cuales fueron muy utilizadas en los países comunistas de la Europa Central y del Este. Los últimos dos métodos tuvieron sorprendentes similitudes con el desarrollo de la terapia behaviorista y las terapias cognitivas en el Oeste. De alguna manera ésto fue así, porque a ambos lados de la ‘’cortina de hierro’’ los profesionales se inspiraron en las tradiciones europeas compartidas desde finales del siglo XIX y principios del siglo XX; sin embargo, este artículo argumenta que dentro de la esfera soviética aquéllos que promovieron estos métodos se apropiaron del pensamiento socialista como fuente de inspiración y justificación o al menos como un escudo político conveniente.

## Abstract (it)

Questo articolo tratteggia ciò che la ricerca recente e le fonti principali dicono della psicoterapia nell'Europa comunista e di come essa sia sopravvissuta sia in modo nascosto sia in modo più evidente. Inoltre, vengono approfondite le tecniche psicoterapeutiche più popolari nei diversi paesi e culture della sfera sovietica, con particolare attenzione al periodo della Guerra Fredda. L’articolo esamina quindi la letteratura sulle fortune alterne della psicoanalisi e delle terapie di gruppo in questa area geografica. Più specificamente, si focalizza su alcune prassi terapeutiche come la terapia del lavoro, la suggestione e la terapia razionale che hanno avuto ampia popolarità nei paesi comunisti dell'Europa centrale e orientale. Gli ultimi due approcci presentano sorprendenti somiglianze con le terapie comportamentali e cognitive che, in parallelo, si andavano sviluppando in Occidente. Ciò era anche dovuto al fatto che i clinici di entrambe le parti della "cortina di ferro" attingevano da tradizioni europee condivise tra la fine del XIX e l'inizio del XX secolo. Tuttavia, questo articolo sostiene che nella sfera sovietica, coloro che promuovevano questi approcci si appropriavano del pensiero socialista come fonte di ispirazione e giustificazione, o per lo meno come un conveniente scudo politico.

## Abstract (fr)

Cet article retrace ce que les recherches récentes et les sources principales nous disent au sujet de la psychothérapie dans l’Europe communiste et comment elle a survécu à la fois de manière souterraine et en surface. Je mettrai l’accent, en particulier, sur les techniques psychothérapeutiques qui étaient populaires dans les différents pays et cultures langagières de la sphère soviétique avec une référence plus spécifique à la période de la guerre froide. Cet article examine la littérature portant sur les résultats mitigés de la psychanalyse et des thérapies de groupe dans cette région. Il se concentre plus spécifiquement sur les modalités thérapeutiques telles que la thérapie par le travail, la suggestion et la thérapie rationnelle, qui furent très populaires dans les pays communistes d’Europe Centrale et de l’Est. Les deux dernières modalités présentent des ressemblances étonnantes avec les développements des thérapies comportementales et cognitives de l’Ouest. Ceci est dû en partie au fait que les cliniciens des deux côtés du rideau de fer se sont inspirés de traditions européennes communes datant de la fin du 19ieme, début du 20ieme siècle. Cependant cet article soutient que dans la sphère soviétique, ceux qui promouvaient ces approches s’appropriaient la pensée socialiste en tant que source d’inspiration et de justification, ou tout du moins, en faisait le bouclier politique idéal.

## Abstract (el)

Περίληψη: Αυτό το άρθρο εστιάζει στις πρόσφατες έρευνες και στις πρωταρχικές πηγές για την ψυχοθεραπεία στην Κομμουνιστική Ευρώπη και το πώς επιβίωσε τόσο κάτω όσο και πάνω από την επιφάνεια. Συγκεκριμένα, θα αναπτύξω τις ψυχοθεραπευτικές τεχνικές που ήταν δημοφιλείς στις διαφορετικές χώρες και γλωσσικές κουλτούρες που βρισκόταν κάτω από τη σφαίρα επιρροής της Σοβιετικής Ένωσης, με ιδιαίτερη εστίαση στην περίοδο του Ψυχρού Πολέμου. Αυτό το άρθρο εξετάζει την βιβλιογραφία που αφορά την ανάμικτη μοίρα που είχε η ψυχανάλυση και η ομαδική θεραπεία σε αυτές τις περιοχές. Πιο συγκεκριμένα, εστιάζει σε θεραπευτικές πρακτικές όπως είναι η θεραπεία μέσω εργασίας, η θεραπεία βάσει υποβολής και λογικής, που έγιναν ιδιαίτερα δημοφιλείς στις Κομμουνιστικές χώρες της Κεντρικής Ανατολικής Ευρώπης. Οι δύο τελευταίες προσεγγίσεις είχαν εντυπωσιακές ομοιότητες με την παράλληλη ανάπτυξη των συμπεριφορικών και γνωστικών θεραπειών στη Δύση. Εν μέρει, αυτό συνέβη γιατί οι κλινικοί και στις δύο πλευρές του «σιδηρούντος παραπετάσματος» άντλησαν από κοινές ευρωπαϊκές παραδόσεις από τα τέλη του 19^ου^ και τις αρχές του 20^ου^ αιώνα. Ωστόσο, το παρόν άρθρο υποστηρίζει ότι στη Σοβιετική Ένωση, αυτοί που προώθησαν αυτές τις προσεγγίσεις καπηλεύτηκαν τη σοσιαλιστική σκέψη ως πηγή έμπνευσης και δικαιολόγησης, ή τουλάχιστον, ως μια βολική πολιτική ασπίδα.

What happened to psychotherapy behind the so-called Iron Curtain? Some commentators have assumed that it was almost non-existent in the Soviet sphere, only to emerge as a significant force in society after the fall of the Berlin Wall (Rose, 1991). Others have written of the vanishing of the unconscious in Eastern Europe, after Freud’s work was officially banned by the Communist authorities (Segal, 1975; Wortis, 1953). More recently, these generalisations have been questioned by historians who have begun to mine archives, and conduct interviews with clinicians from the ‘psy’ professions who practised during the Communist period (Dufaud, 2011; Eghigian, 2002; Eghigian, Killan, & Leuenberger, 2007; Marks & Savelli, 2015; Zajicek, 2009, 2014). This research has unveiled a miscellany of therapeutic approaches, some of which sought to build on socialist philosophies of mind and human nature, and others which positively eschewed Marxism altogether. In this article, I will trace what these contributions, along with primary sources, can tell us about the state of psychotherapy in Communist Europe, and how it survived both underground and above the surface. In particular, I will elaborate on the psychotherapies that were popular across the different countries and language cultures of the Soviet sphere, with a particular focus upon the Cold War period. This article examines the literature on the mixed fortunes of psychoanalysis and group therapies. More specifically, it reconstructs therapeutic approaches such as suggestion, rational therapy and work therapy, which, in some part, looked to socialist thought as a source of inspiration or, at the very least, as a convenient political shield. Drawing from approaches in the Sociology of Scientific Knowledge (Bloor, 1976/1991), this article does not seek to make normative claims about the psychotherapeutic techniques described, nor does it take a stance in relation to the ethics or politics of the Soviet Union and its satellite countries. Instead, its focuses on exploring the way in which particular modes of treatment survived, or in some cases became ascendant, and the types of justification that were used to support them in the historical context of Communist Europe.

## Psychoanalysis: Underground and in plain sight

The sometimes paradoxical fate of psychoanalysis under Communism has been a source of particular fascination for historians and practitioners alike. In the Soviet Union, the initial years after the Bolshevik Revolution saw a surprising level of tolerance and state-sponsored experimentation with Freudian concepts. Accusations of idealism were levelled by Marxist–Leninist philosophers in the critical literature in the 1920s, but for a number of years after 1917, theories of the unconscious and childhood sexuality were not, in fact, seen to be extraneous to the revolutionary project (Etkind, 1997; Launer, 2015; Miller, 1998; Proctor, 2016). The most striking case study is the Children’s House (*Detski Dom*) experiment in Moscow, a therapeutic residential institution for children led by the psychoanalytic educationalist Vera Schmidt. With many attendees from among the children of the Party elite, including Stalin’s own son, the Children’s House pioneered psychoanalytic child rearing practices with involvement from Sabina Spielrein and Alexander Luria (Miller, 1998; Schmidt, 1924; Valkanova, 2016). The project was short-lived, however, with Stalin opting to close it as a demonstration of opposition against Trotsky’s vocal support for psychoanalysis. The existence of the Children’s House should be considered within the broader context of post-revolutionary debates about the reform of the family, communal living and the collective responsibility for child rearing (Miller, 1998). (And it also, later, provided a source of inspiration for Wilhelm Reich and radical student movements in 1968) (Valkanova, 2016).

Stalin’s ascent to power after the death of Lenin in 1924 saw a radical revision of policy, with psychoanalysis officially blacklisted, and Pavlovian techniques elevated in status as the politically acceptable form of approach in the clinical ‘psy’ disciplines by the 1950s (Zajicek, 2009). In the countries of Central and Eastern Europe, which came under Communist control in the late 1940s, a number of psychoanalytic circles, and even officially recognised local branches of the International Psychoanalytical Association, had coalesced by the time of the outbreak of war in 1939. Their growth was in part facilitated by their geographical proximity to Vienna, the birthplace and the main geographical locus of psychoanalysis throughout the inter-war period. These communities suffered significant losses during the war, with many of their number losing their lives in the Nazi concentration camps. Others went into exile in the West, with a cohort going on to become key players in building the psychoanalytic movement in the US (Erös, 2016). But some did survive, and remain, in the new People’s Democracies of the region, and continued to train and practice in unofficial settings, often behind the closed doors of the asylum, or in individuals’ private homes (Kovai, 2015; Leuenberger, 2001; Marks, 2015; Savelli, 2013).

Psychoanalysis also continued to inform practice within institutions, sometimes because mental health was considered sufficiently low down on the political priority list that clinicians were allowed to continue their work unnoticed by the authorities, as in the case of Hungary (Kovai, 2015). In East Germany, many made a deliberate choice to reframe their psychoanalytic practice in different terminology, masking research and practice which was otherwise conceptually unchanged (Leuenberger, 2001). In Czechoslovakia, there was a dramatic innovation and expansion of psychoanalytically informed therapies with state funding, through a nationally supported psychedelic psychiatry project which ran from the mid 1950s through to 1974, primarily using LSD to induce abreaction and accelerate the therapeutic process (Crockford, 2007; Marks, 2015). The material presented by patients in these sessions, sometimes mediated through art therapy, was often interpreted as a manifestation of childhood trauma, but also through frameworks informed by Otto Rank’s writings on perinatal experience and birth trauma, the Jungian collective unconscious or even the phylogenetic unconscious reverting back to previous evolutionary states, drawing from Carl Jung and Sándor Ferenczi. These approaches had a striking legacy in the international development of transpersonal psychotherapies, with one of the key proponents, Stanislav Grof, emigrating to the USA in 1967 and continuing his work there, largely shaped by the experiences of the Prague-based projects (Marks, 2015).

In Yugoslavia, a Communist country that remained independent of the Soviet sphere of control, psychoanalysis had unexpected fortunes within state-run facilities (Savelli, 2013). Historian Ana Antic has also argued that analytic concepts came to play a particular role in rehabilitation programmes. There was a congruence, she argues, between the goals of both psychoanalysis and the Yugoslav self-management style of socialism, in terms of reforming individuals by prompting them to re-script their life stories according to a template. In the case of Tito’s prison camps at Goli Otok, where psychologists informed the re-education programmes, these techniques crossed the line from therapy to coercion (Antic, 2016a, 2016b).

## Work therapies

Work therapy, as one might imagine, came to have special place in Marxist–Leninist oriented societies, with the emphasis they placed upon work as central to healthy human functioning, and the importance of the contribution of the individual to the social collective. That said, the role of Communism in facilitating its emergence as a therapeutic modality in Russia and Eastern Europe should not be overestimated. In many countries, work therapies had already become a routine feature of life in asylums and sanatoria the region, as in much of the rest of Europe, from the nineteenth century. ‘Moral Treatment’, invented at the York Retreat in the 1790s, gradually grew in popularity elsewhere, often in the form of agricultural therapies carried out in the grounds of institutions. In the lands of Germany and the Habsburg Empire, these approaches to treatment were explicitly incorporated into the design of a number of psychiatric institutions – sometimes designed by prestigious modern architects – by the *fin de siècle* (Engstrom, 2011; Topp, 2011, 2017).

In Russia, too, work therapy predated the 1917 Revolution. During the Soviet period, there were a number of schemes for the employment of psychiatric patients in agricultural colonies and in turbine factories built within the grounds of asylums, where payment in return for labour was a possibility, and patients could join trade unions (Sirotkina & Kokorina, 2015). While work therapy was not an invention of the Communist state, as Irina Sirotkina and Marina Kokorina have shown, it nevertheless withered away with the collapse of the Soviet Union (2015). While there were contingent reasons for its disappearance, the lack of a collective will to lobby for its continuation is also telling of a move away from institutionalised work therapy internationally by the end of the twentieth century. It also signalled the shift in the ideological landscape of Russia itself. There was no longer any significant investment in the idea that employment should guaranteed by the state. Nor in the belief that labour was fundamental to the positive shaping of the individual’s personality, ultimately enabling society to function as a whole.

## The group and the collective

Work therapies may have been the obvious candidate to focus on in the search for a ‘socialist’ psychotherapy. But there are others, too, which have been isolated for examination by historians on the supposition that they may have a particularly political character: most especially group therapies. The primacy of collectivism, somewhat predictably, came to be invoked as a way of justifying and promoting these approaches at times. But we need to be careful not to retrospectively overemphasise the significance of groups, as they by no means came to replace – or even to surpass – individual psychotherapies formed around the patient–practitioner dyad. For Miassischev, one of the key ideologues of Soviet psychotherapy, group approaches were useful primarily as a means of reinforcing the effects of treatments that had already been carried out on an individual basis. Cases could be discussed, and patients with similar pathologies could mutually support each other in group-based sessions, in such a way as to ‘normalise social relations’ (Misassischev, 1960, p. 19, 20). However, Miassischev stopped short of asserting that the collective nature of the group might itself have therapeutic agency, or even that it could be important as a way of simulating socialist society on a smaller scale. He even added a caveat, a warning that group therapy was flawed because of its ‘dependence on Freud’ (p. 20).

By contrast, in East Germany, group psychotherapeutic practice was indeed framed more explicitly as being in tune with the goals of socialism, with innovations emerging as a consequence of these imperatives. Christine Leuenberger has charted the development of Intentional Dynamic Group Psychotherapy by Kurt Höck at the Berlin psychotherapeutic unit in the 1970s, not long after it was threatened with closure due to the time and expense taken up by the provision of individual approaches (Geyer, 2011; Leuenberger, 2001, p. 265). The group ‘dynamic’ was exploited as a therapeutic force in itself: while the therapist guided the patients towards an ‘intended’ goal, they themselves were merely a facilitator. Placing the focus on the interpersonal, socio-psychological processes which played out within this collective setting, individuals were, in Leuenberger’s words, ‘retrained to act meaningfully in society … and generally strengthened so they could partake in industrial production’ (p. 265).

Such justifications for the ideological validity of therapies were indeed appealing to clinics and medical schools, who were very much aware of being under the watchful eye of the state. Yet, as Mat Savelli argues for the Yugoslav case, many of the clinicians who established group therapy techniques were motivated by an allegiance to social psychiatry as an approach, rather than socialism as such. Inspiration was often drawn from international sources, including the UK-based analyst S.H. Foulkes, with some therapists having also spent time at the Tavistock Clinic in London (Savelli, in press). The striking popularity of Jacob Moreno’s ‘psychodrama’ across the East European region also testifies to an open-mindedness towards the provenance of techniques. This theatre-based approach, which encouraged individuals to literally act out, and then reflect upon, their emotional conflicts in relation to other group participants, was developed primarily in the United States (Aleksandrowicz, 2009; Kratochvíl, 1977; Lauterbach, 1984; Moreno, 2014). Sources from Czechoslovakia in the later years of socialism also show that an eclectic range of authors from both East and West were familiar. Here, Wilfred Bion and Carl Rogers were just as likely to appear in reference lists as key Soviet authors such as Miassischev, Rozhnov or Makarenko (Kratochvíl, 1977). All of these authors, regardless of origin, were taken seriously as sources of inspiration. Group approaches certainly had their place in the psychotherapeutic armament in the Communist East – much as in the West – but, perhaps somewhat surprisingly, they were rarely theorised in terms of Marxist collectivism.

## Suggestion

The dry and pedantic utterances of a tired physician will not cure a single patient. But suggestions – disturbing, arousing, inspiring suggestions – represent a complex and dynamic system of words and meanings, imagery and motions, as well as a functionally psychological and, consequently, physiological totality capable of combining a dynamic form and a significant content. (Misassischev, 1960)

Suggestion, in various forms, was probably the most widely practiced form of psychotherapy in the Soviet sphere of influence (Aleksandrowicz, 2009; Geyer, 2011; Kondáš, 1997; Winn, 1960). Imported to Russia in the early twentieth century by physicians who had made the pilgrimage to prestigious medical institutions in France and Germany for training, suggestion in waking states, as well as through hypnosis, enchanted both clinical and popular audiences in the 1900s (Sirotkina, 2001, p. 102).

Conditioned reflexes also became a central focus of research of some of the most celebrated scientists in the Soviet Union: Vladimir Bekhterev, Ivan Pavlov and Konstantin Bykov. By 1951, the work of Pavlov and his associates were the only officially sanctioned conceptual basis for medicine and the psy-disciplines in the Soviet Union, rendering suggestion-based therapies the most secure approach to work with in political and economic terms (Zajicek, 2009). A canonical text, Konstantin Platonov’s *The Word as a Physiological and Therapeutic Factor: The Theory and Practice of Psychotherapy According to I.P. Pavlov*, was published in Russian in 1957, and translated into English by the Moscow Foreign Languages Publishing House in 1959 (Platonov, 1959). Drawing from a wealth of experimentation carried out in the Soviet Union, Platonov made the case for the fundamental role of a particular form of talking therapy. Pavlov had shown, he argued, that humans shared the same type of higher nervous activity as the rest of the animal kingdom. Man, however, had ‘a special socially conditioned addition which shows a qualitative peculiarity. This addition is connected with labour and social activity, concerns the speech function and introduces a new principle into the activity of the cerebral hemispheres’ (p. 16).

Laboratory research, according to Platonov, had shown that the utterance of words was ‘far from immaterial to the human organism’, and could have profound effects on physiology (p. 17). Investigations had, for example, demonstrated that it was the very meaning of the word, rather than its sound, which produced an effect upon the body (Shvarts, 1948, 1949). For example, when a person’s skin was pricked by a pin, there was an effect on their blood pressure – a similar, if slightly less marked effect could be generated in response to the utterance of the *word* ‘pinprick’ (Platonov, 1959, p. 21). While these effects were complex, Platonov and others suggested it was feasible to extrapolate from these findings. Words, they argued, had been shown to have myriad effects on both brain and body, and suggestion could therefore be harnessed for medical purposes. Furthermore, it had the potential to treat all organs of the body, in addition to the neurotic disorders more traditionally associated with psychotherapy (Platonov, 1959; Rozhnov, 1954). While the word ‘psychotherapy’ was retained, these practices went far beyond the treatment of the psyche alone, becoming applied to a range of somatic ailments.

In East Germany, at the university polyclinic in Jena, similar ‘autogenic relaxation’ techniques were also pioneered by two internal medicine specialists, Gerhard Klumbies and Helmuth Kleinsorge, who had taken over the psychotherapeutic clinic after the war due to staff shortages. Instead of looking back to Russian and French research, they drew upon the work of Jena’s own Johannes Schultz and local German psychosomatic traditions from the pre-war period. The clinic became a centre for both experimentation and training: it hosted workshops for the instruction of physicians and therapists from across the Eastern bloc countries, and some from the West (Geyer, 2011; Kleinsorge & Klumbies, 1962). The Jena clinic also produced vinyl records for the purposes of training individuals in self-hypnosis, with booklets demonstrating positions and breathing exercises (Kleinsorge & Klumbies, 1962). Here, we see how psychotherapy left the clinic in the socialist world, forming the basis of prophylactic practices that could be disseminated widely, and carried out in private. This focus on suggestion for prevention and the positive reinforcement of desired behaviours was a key aspect of psychotherapy under socialism. As an example of the utopian uses of psychotherapy, Aleksandra Brokman has shown how autosuggestion came to be used to train Soviet athletes, illustrating the imperative to use these techniques to shape thoughts and emotions in the interests of ‘self perfection’, far beyond the context of the hospital and clinic (Brokman, 2017).

## Persuasion and rationality

In 1960, the Russian psychotherapist Miassischev claimed that there were two main approaches to therapy in the Soviet world: ‘suggestion and persuasion. They differ mainly in the purpose of the activating words. Sometimes they are contrasted as the irrational and the rational’ (Misassischev, 1960). The latter – usually described simply as ‘rational therapy’ – was based, in Pavlovian terms, on the most sophisticated functions of the ‘secondary signalling system’, the parts of the higher nervous system that were specific to the human species. Through active engagement with these conscious, critical faculties based on the capacity for language, it was believed that patients could be literally persuaded back into sound reasoning, and better mental and physical health. While it was developed most enthusiastically in Soviet Russia, rational therapy was taken up in clinics across the region, especially in Poland and Czechoslovakia (Aleksandrowicz, 2009; Kondáš, 1997).

As was the case for work therapies and suggestion, rational therapy was by no means a Soviet – or even a Russian – invention. It was originally the creation of Paul Dubois, a Swiss medical doctor who practiced in Berne in the early twentieth century. Dubois’ method was formulated in direct opposition to suggestion, on ethical grounds, arguing that it gave too much power to the therapist at the expense of the patient’s own agency (Dubois, 1908; Müller, 2001; Shamdasani, 2005, p. 10). By strengthening the individual’s innate ability for logical reasoning, Dubois argued, they could regain not only a sense of healthy functioning, but also a restoration of their personal autonomy.

Whilst largely forgotten in the West, in the early years of the twentieth century, rational therapy was a worthy competitor to psychoanalysis in terms of its popularity in Europe, and this extended as far Russia. There, its status did not, in fact, diminish to the extent that it had elsewhere by the mid-century (Sirotkina, 2001). Rational therapy, like suggestion, was another technique whose long-term fortunes in the Soviet sphere were considerably more favourable than in its own region of origin. In part, one could account for this as the result of the demise of psychoanalysis as an officially sanctioned approach in the USSR after the mid-1920s (Angelini, 2008). Without such a vociferous and institutionally robust rival in Russia, other psychotherapeutics had more space to develop.

But there were also philosophical reasons underlying the enduring regard for rational therapy. The predominant theory of mind in the Soviet Union, beyond Pavlovian studies of higher nervous activity, was Lenin’s ‘theory of reflection’. First elaborated in his book *Materialism and Empirio*-*Criticism*, Lenin asserted that the mind was able to form a reflection of independent, external world. The accuracy of this reflection was bolstered by the findings of science and logical deduction, but it could never be a flawless, unmediated representation of reality, always remaining an ongoing, dialectical process of best approximations. Nevertheless, without ever attaining a fully exact knowledge of the surrounding world, human beings, according to Lenin, had the capacity to achieve a reliable, working reflection of it (Lenin, 1909).

This conception placed a high value on the capacity for scientific reasoning as an essential feature of what it meant to be human: empirical rationality was fundamental to the Soviet model of the healthy mind. Theoreticians of psychotherapy in the Communist period skilfully appropriated the theory of reflection to explain the underlying processes at play in rational therapy. Mental illness was construed as a the consequence of a fault having occurred in the internal reflection of external reality: through a careful process of demonstrating to the patient how these thoughts or assumptions were not, in fact, supported by empirical evidence, the patient could be fundamentally reasoned out of their neurosis (Lauterbach, 1984).

There are some remarkable similarities between the practice of rational therapy in the East, and Albert Ellis’s Rational Emotive Behaviour Therapy, or indeed the closely related Cognitive Therapy techniques, developed in the United States from the 1960s, despite knowledge exchange not having occurred across East and West on these particular matters (Beck, 1979; Ellis, 1971). More specifically, REBT’s deference to Stoic philosophy, especially Epictetus’ epithet that ‘men are not disturbed by things, but the views which they take of them’, was explicitly mirrored in Soviet rational therapy debates (Dryden & Still, 2012; Lauterbach, 1984). Ellis also, notably, took retrospective inspiration from Dubois to further develop his techniques, indicating that the latter’s work has had more of a legacy than historians have acknowledged (Dryden & Still, 2012). That psychotherapeutic techniques should have come to develop in parallel with each other to such an extent, across the so-called Iron Curtain, may appear counterintuitive. But it can be read as testament to the similar imperatives on both sides for the creation of self-controlled persons, guided by enlightenment ideals of rationality, at the very height of Cold War modernity (see Krylova, 2014).

## Conclusions

The fall of the Berlin Wall and the Collapse of the Soviet Union shifted the terrain for the role of psychotherapy in the region. In some post-socialist countries, psychotherapeutic concepts have come to play a new type of function in social life, becoming taken up as a way of trying to work through, and come to terms with, difficult aspects of the Communist past – at both a personal and a national, collective level (Leuenberger, 2000; Marks, 2017). It has also certainly been the case that the collapse of Communism enabled an expansion of psychotherapeutic practice, both in terms of the quantity of professionals offering therapy, and the variety of approaches available in private practice (Rose, 1991). But this isn’t, of course, to say that the psychotherapeutic professions, and the knowledge they produced, were absent, or did not hold a stake in society under socialism (Buda, Tomcsanyi, Harmatta, Csaky-Pallavicini, & Paneth, 2009; Calloway, 1993; Doboş, 2015; Raikhel & Bemme, 2016).

Adéla Gjuričová, writing on the last two decades of the Communist regime in Czechoslovakia, reminds us that some forms of therapy existed with norms of private payment for years before the emergence of an official free market economy, suggesting that these forms of transaction might have paved the way for the acceptance and proliferation of private practice in the 1990s (Gjuričová, in press). As noted above, a plurality of techniques – including some that the state was officially hostile towards – existed both underground and semi-officially in most countries, even if those practising them were only afforded limited degrees of freedom. But there are other distinctive continuities between the socialist and post-socialist periods. The clear resonances between Pavlovian and suggestion-based therapies with behavioural approaches, as well as between rational therapies and cognitive approaches, meant that clinicians trained in these techniques were able to rebrand themselves as cognitive and behavioural therapists in the 1990s, with some even framing the former as ‘predecessors’ to the latter in their own histories of the profession (Kondáš, 1997).

It is also important not to underplay the continuities and similarities in practice across East and West, in some cases facilitated by exchange of knowledge and personnel across borders. In other instances, this was more a legacy of pre-Communist, trans-European intellectual networks, especially engagement with French and German language cultures, dating back to the late nineteenth and early twentieth centuries. And yet, for certain approaches, particularly those based on suggestion, rational persuasion, or the value of work, the socialist context – and the philosophies fostered by the Soviet Union and its satellites – did tangibly shape the development of therapeutic theory and practice. For all of the limitations and restrictions places on intellectual freedom within the Soviet sphere, in these cases, socialism was itself a muse for innovation.

## Disclosure statement

No potential conflict of interest was reported by the author.

## Funding

This work was supported by the Wellcome Trust [grant number 103344/Z/13/Z].

## Notes on contributor

***Sarah Marks*** is a postdoctoral researcher at Birkbeck, University of London. Her research focuses on the history of the psy-disciplines during the cold war. Her recent publications include *Psychiatry in Communist Europe* (co-edited with Mat Savelli, Palgrave, 2015) and a special issue of *History of the Human Sciences* on ‘Psychotherapy in Historical Perspective’ (April 2017).
